# Prognostic value of baseline FDG PET/CT in HER2-positive metastatic breast cancer treated with first-line trastuzumab, pertuzumab, and docetaxel

**DOI:** 10.1186/s13058-025-02191-7

**Published:** 2025-12-10

**Authors:** Marcin Kubeczko, Andrea d’Amico, Olgierd Chrabanski, Daria Handkiewicz-Junak, Anna Polakiewicz-Gilowska, Katarzyna Swiderska, Marta Mianowska-Malec, Aleksandra Lesniak, Barbarba Lanoszka, Natalya Lisovska, Damian Borys, Bartlomiej Pycinski, Ewa Chmielik, Slawomir Blamek, Aleix Prat, Michal Jarzab

**Affiliations:** 1 Breast Cancer Center, Maria Sklodowska-Curie National Research Institute of Oncology, Gliwice Branch, 15 Wybrzeze Armii Krajowej Street, 44-102 Gliwice, Upper Silesia Poland; 2 Department of Nuclear Medicine and Endocrine Oncology, Maria Sklodowska-Curie National Research Institute of Oncology, 44-102 Gliwice, Poland; 3 Tumor Pathology Department, Maria Sklodowska-Curie National Research Institute of Oncology, 44-102 Gliwice, Poland; 4 Faculty of Biomedical Engineering, Silesian University of Technology, Roosevelta 40, 41-800 Zabrze, Poland; 5 Department of Radiotherapy, Maria Sklodowska-Curie National Research Institute of Oncology, 44-102 Gliwice, Poland; 6 Translational Genomics and Targeted Therapies in Solid Tumors, August Pi I Sunyer Biomedical Research Institute (IDIBAPS), Barcelona, Spain; 7 Medicine Department, University of Barcelona, Barcelona, Spain; 8 Cancer Institute and Blood Disorders, Hospital Clinic, Barcelona, Spain; 9 Reveal Genomics, Barcelona, Spain

**Keywords:** FDG PET/CT, HER2-positive metastatic breast cancer, SUVmax, Trastuzumab-pertuzumab-docetaxel, Biomarker

## Abstract

**Background:**

Trastuzumab, pertuzumab, and docetaxel (THP) have represented the standard first-line treatment for HER2-positive metastatic breast cancer (MBC) for over a decade, following the pivotal CLEOPATRA trial. However, interim results from the DESTINY-Breast09 trial suggest that trastuzumab deruxtecan (T-DXd) may soon replace THP as the new first-line standard. Recent developments in treatment have raised new questions about how best to match therapy intensity with individual patient profiles. FDG PET/CT, routinely used for metabolic imaging, might help by identifying patients with differing prognoses based on baseline tumor activity. We investigated the prognostic relevance of baseline FDG PET/CT and its association with treatment outcomes in HER2-positive MBC patients receiving first-line THP.

**Methods:**

We retrospectively analyzed 194 patients with HER2-positive MBC who underwent baseline FDG PET/CT within 12 months before initiating THP therapy (2017–2024). SUVmax was recorded, and optimal cut-off values for progression-free survival (PFS) was determined by ROC analysis. Cox regression and logistic regression were used to identify independent predictors of PFS, OS, and long-term response (PFS ≥ 35 months).

**Results:**

Median PFS was 29.1 months; 2-year PFS was 60.3%. SUVmax > 9.6 was associated with significantly shorter PFS (18.9 vs. 43.9 months; *p* = 0.002) and remained independently prognostic in multivariate analysis (HR 1.98; *p* = 0.001). Median OS was 85.0 months. Among 162 evaluable patients, 35% were classified as long responders. ER positivity (OR 4.82), oligometastatic disease (OR 2.60), and low SUVmax (≤ 9.6; OR 2.78) were independent predictors of durable benefit.

**Conclusions:**

Baseline SUVmax derived from FDG PET/CT is an independent predictor of PFS in HER2-positive MBC treated with first-line THP. Integration of metabolic imaging biomarkers with clinical and molecular factors may support more personalized treatment approaches in this evolving therapeutic setting.

**Supplementary Information:**

The online version contains supplementary material available at 10.1186/s13058-025-02191-7.

## Introduction

Over the past two decades, HER2-targeted therapy has dramatically improved outcomes in advanced HER2-positive breast cancer [1–3]. Trastuzumab combined with chemotherapy was the initial backbone, and the landmark phase III CLEOPATRA trial established that adding pertuzumab to first-line trastuzumab plus docetaxel (THP regimen) confers a significant survival advantage [4]. In CLEOPATRA, dual HER2 blockade with THP prolonged median progression-free survival (PFS) from 12.4 to 18.5 months, and subsequent analyses showed a remarkable median overall survival (OS) of 56.5 months with THP versus 40.8 months with chemotherapy and trastuzumab alone [5]. These results revolutionized the standard of care (SOC), with THP becoming the first-line regimen of choice since 2012. While THP remains an effective first-line option, many patients ultimately relapse or fail to achieve sustained disease control. This underscores the importance of developing new approaches that can both prolong therapeutic benefit and enable more tailored, biomarker-driven treatment decisions.

One of the most promising developments in HER2-targeted therapy is trastuzumab deruxtecan (T-DXd), an antibody–drug conjugate that has demonstrated impressive efficacy in the second-line setting [6] The DESTINY-Breast03 trial confirmed its superiority over T-DM1 in previously treated HER2-positive MBC [6]. Building on this, the ongoing phase III DESTINY-Breast09 trial is evaluating T-DXd in the first-line setting, randomizing patients to receive standard THP, T-DXd plus pertuzumab, or T-DXd monotherapy [7]. Interim results presented at ASCO 2025 showed that T-DXd combined with pertuzumab significantly improved median PFS compared to THP (40.7 vs. 26.9 months), raising the possibility that this combination could replace THP as the standard first-line regimen [7]. These findings are promising, but they also point to the need for more individualized treatment strategies. Patients differ in their response to standard therapy, and being able to identify those who may benefit from intensified or more targeted approaches is becoming increasingly important. Several biomarker-defined subgroups have been explored in this context.

Patients with hormone receptor-positive (HR+)/HER2+ MBC may exhibit distinct resistance mechanisms. The AFT-38 PATINA trial evaluated palbociclib added to maintenance anti-HER2 and endocrine therapy, demonstrating a clinically meaningful improvement in PFS (44.3 vs. 29.1 months). This suggests that CDK4/6 inhibition may overcome endocrine resistance in this subgroup [8]. In parallel, central nervous system (CNS) involvement affects up to 50% of patients with HER2 + MBC. Tucatinib, a HER2-selective TKI with CNS activity, improved both PFS and OS in the HER2CLIMB trial [9], especially in patients with active brain metastases [10]. The ongoing HER2CLIMB-05 study is assessing whether adding tucatinib to trastuzumab and pertuzumab in the maintenance setting can delay or prevent CNS progression [11]. Beyond hormone receptor status and CNS involvement, genomic alterations such as PIK3CA mutations represent another biologically relevant factor associated with treatment resistance. Present in 22–32% of HER2+ tumors, these mutations activate the PI3K/AKT/mTOR pathway and confer inferior outcomes with anti-HER2 therapy [12, 13]. In the CLEOPATRA trial, patients with these mutations had shorter PFS despite dual blockade [13]. The INAVO122 trial is testing inavolisib, a PI3Kα-selective inhibitor [14], in combination with trastuzumab and pertuzumab for PIK3CA-mutant HER2+ MBC, aiming to establish a new biomarker-driven approach for this high-risk population [15].

The clinical challenge of selecting the most appropriate treatment strategy for patients with HER2-positive breast cancer is becoming increasingly complex, particularly in the metastatic setting. Determining which of these aforementioned modifications is optimal for a given patient—especially in the absence of overt clinical progression—remains a major clinical and research question. The challenge is further amplified by the practical limitations of testing all possible combinations across the broad spectrum of clinical scenarios. Currently, HER2 overexpression remains the primary predictive biomarker guiding anti-HER2 therapy. However, aside from HER2 itself, there is a critical lack of validated biomarkers that can reliably predict benefit or resistance to specific anti-HER2 agents [16]. This underscores the urgent need for additional biological or functional markers to facilitate more refined patient stratification and treatment personalization.

Functional imaging with 2-[¹⁸F]fluoro-2-deoxy-D-glucose positron emission tomography/computed tomography (FDG PET/CT) is increasingly recognized as a key diagnostic and prognostic tool in breast cancer. Unlike conventional imaging modalities such as contrast-enhanced CT (CE-CT), FDG PET/CT provides metabolic insights into tumor biology and disease burden, aiding in the assessment of tumor aggressiveness and heterogeneity—key factors in personalized oncology [17]. Accurate staging is critical, as the presence and extent of metastases directly impact prognosis and treatment. Over the past decade, multiple studies have confirmed the superior diagnostic performance of FDG PET/CT over CE-CT and bone scintigraphy. Reflecting this, joint EANM–SNMMI guidelines—endorsed by ACR, ESSO, ESTRO, EUSOBI/ESR, and EUSOMA—recommend its use for initial staging from stage IIB to IV disease [18].

While conventional imaging may offer advantages in specific anatomical regions, FDG PET/CT has shown superior sensitivity and specificity across most metastatic sites in MBC [19–21]. In a prospective randomized trial comparing FDG PET/CT with standard imaging (bone scan and CT of the chest, abdomen, and pelvis), FDG PET/CT led to upstaging to metastatic disease in 23% of patients, compared to 11% with conventional imaging. This more accurate disease characterization resulted in major treatment modifications, including reduced use of curative-intent multimodal approaches such as surgery and radiotherapy [21]. Furthermore, a 2021 systematic review and meta-analysis highlighted the clinical utility of FDG PET/CT, showing a pooled 25% change in disease staging and an 18% modification in treatment decisions following PET-based evaluation [22]. The modality is particularly effective in detecting bone metastases—a common site of breast cancer spread. FDG PET/CT identified more unequivocal bone lesions than CE-CT or bone scans, altering treatment recommendations in 16% of cases [23]. PET/CT was also found to be superior in detecting lytic and mixed-type lesions, bone marrow involvement [24], and overall bone metastasis burden compared with planar bone scintigraphy [25].

These findings point to a broader role for FDG PET/CT in metastatic breast cancer—not just in staging but also in helping to estimate prognosis. Its ability to combine metabolic and anatomical imaging may make it helpful in identifying patients at higher risk and adjusting treatment intensity accordingly. Thus, we aimed to analyze the prognostic utility of baseline FDG PET/CT in patients with HER2-positive metastatic breast cancer treated with trastuzumab, pertuzumab, and docetaxel in the first-line setting.

## Methods

### Study group

We conducted a retrospective analysis of female patients with HER2-positive metastatic breast cancer (MBC) who received first-line THP (docetaxel, trastuzumab, and pertuzumab) between January 2017 and October 2024. The following inclusion criteria were applied: (1) HER2-positive MBC treated with THP in the first-line setting; (2) age ≥ 18 years; (3) availability of baseline 2-[¹⁸F]FDG PET/CT prior to THP initiation. Patients were excluded if a baseline FDG PET/CT was not available or if it had been performed more than 12 months prior to treatment initiation. The data cut-off for the analysis was June 1, 2025.

### Imaging and response assessment

Baseline imaging was conducted using FDG PET/CT, performed before the initiation of THP. Scans were acquired using either a Gemini XL system (Philips Medical Systems, Eindhoven, The Netherlands) or a Siemens Biograph mCT (Siemens Healthineers, Erlangen, Germany). Imaging protocols followed institutional standards: patients fasted for at least 6 h before intravenous administration of 18 F-FDG (3.7 MBq/kg; total activity range: 185–555 MBq). Scanners were calibrated to ensure accurate dose measurement. Image acquisition began 60 min after tracer injection (± 10 min). The maximum standardized uptake value (SUVmax) was recorded for the lesion with the highest FDG uptake, regardless of its location (i.e., primary breast tumor, regional lymph node, or distant metastasis).

CE-CT was performed at baseline and every 12 weeks thereafter to assess treatment response based on RECIST version 1.1 criteria [26]. OMD was defined as the presence of up to five metastatic lesions.

### Study objectives and endpoints

The primary objective was to evaluate the prognostic utility of baseline SUVmax measured by FDG PET/CT. The primary endpoint was PFS, defined as the time from initiation of THP to disease progression or death from any cause. The secondary endpoint was OS, defined as the time from initiation of THP to death from any cause. We also assessed whether baseline SUVmax was associated with long-term treatment benefit, defining a long response as PFS ≥ 35 months, in line with previous studies.

### Statistical analysis

Categorical variables were summarized as frequencies and percentages. Continuous variables were expressed as mean values with ranges or as medians with interquartile ranges (IQRs, 25th–75th percentile). Differences between groups were evaluated using the Fisher exact test for categorical variables and the Wilcoxon rank-sum test for continuous variables. Time-to-event endpoints (PFS and OS) were estimated using the Kaplan–Meier method, with differences between groups compared using the log-rank test. To identify predictors of PFS and OS, univariate Cox proportional hazards models were used. Variables with a *p*-value < 0.20 in univariate analysis were entered into multivariate Cox models. For categorical variables like ECOG performance status, all categories were included in the analysis, regardless of whether individual levels showed statistical significance. Proportional hazard assumptions were verified for all Cox models. To complement survival analyses, a logistic regression model was developed to assess factors associated with long-term response (PFS ≥ 35 months). Odds ratios (ORs) and corresponding 95% confidence intervals (CIs) were reported. Optimal SUVmax cut-off point for PFS was determined using receiver operating characteristic (ROC) curve analysis and the Youden index [27]. To avoid inconsistencies and multiplicity of thresholds, the same cut-off of was applied for OS analyses. Additionally, an exploratory analysis was performed to calculate the optimal SUVmax cut-off for OS using the same ROC–Youden approach. All statistical tests were two-sided, and a *p*-value < 0.05 was considered statistically significant. Analyses were performed using Stata software (version 18; StataCorp, College Station, TX, USA).

## Results

### Study population

**Fig. 1:**
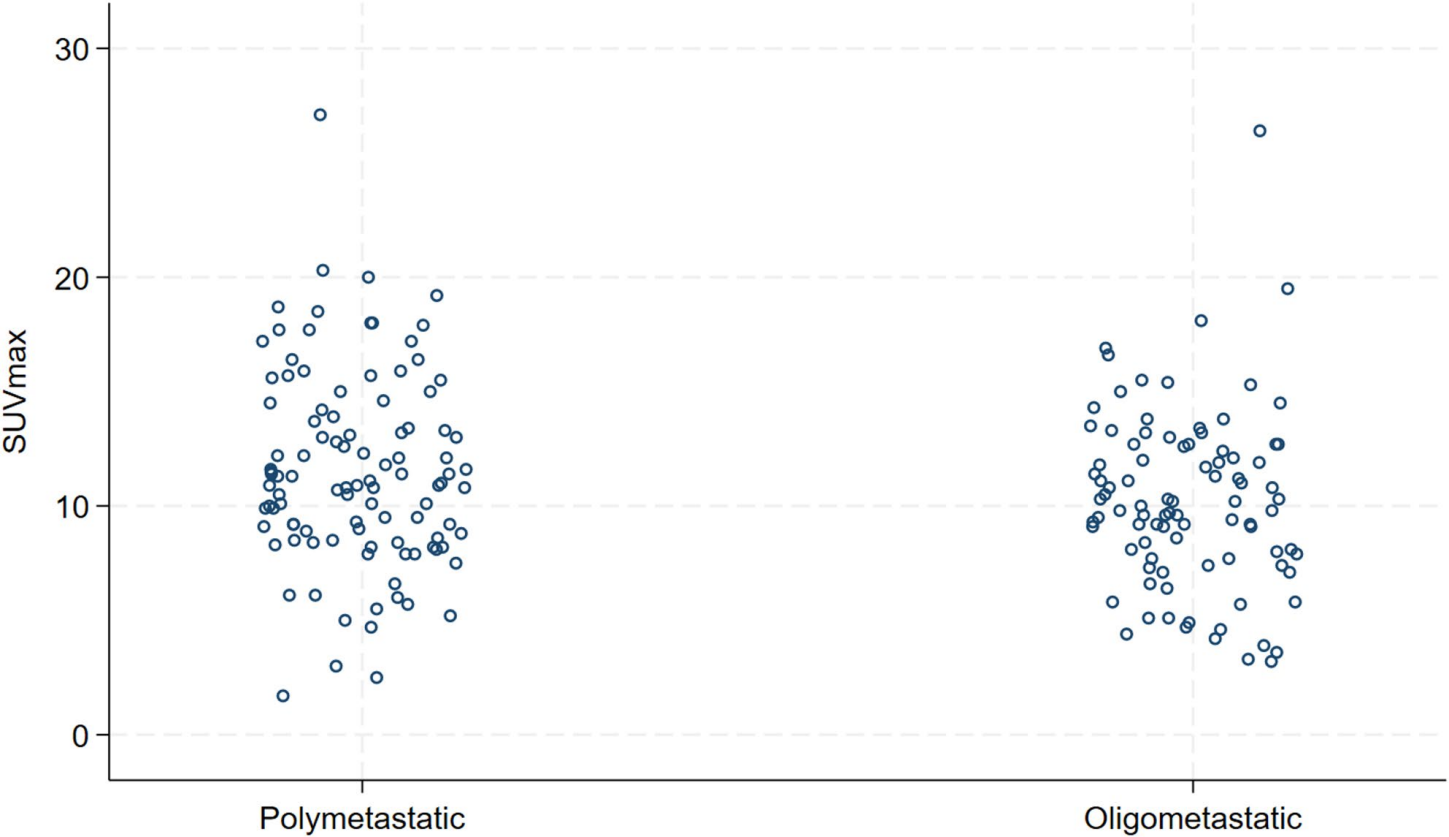
Distribution of baseline SUVmax by disease extent. Each dot represents an individual patient’s SUVmax value, shown separately for polymetastatic and oligometastatic disease

**Fig. 2:**
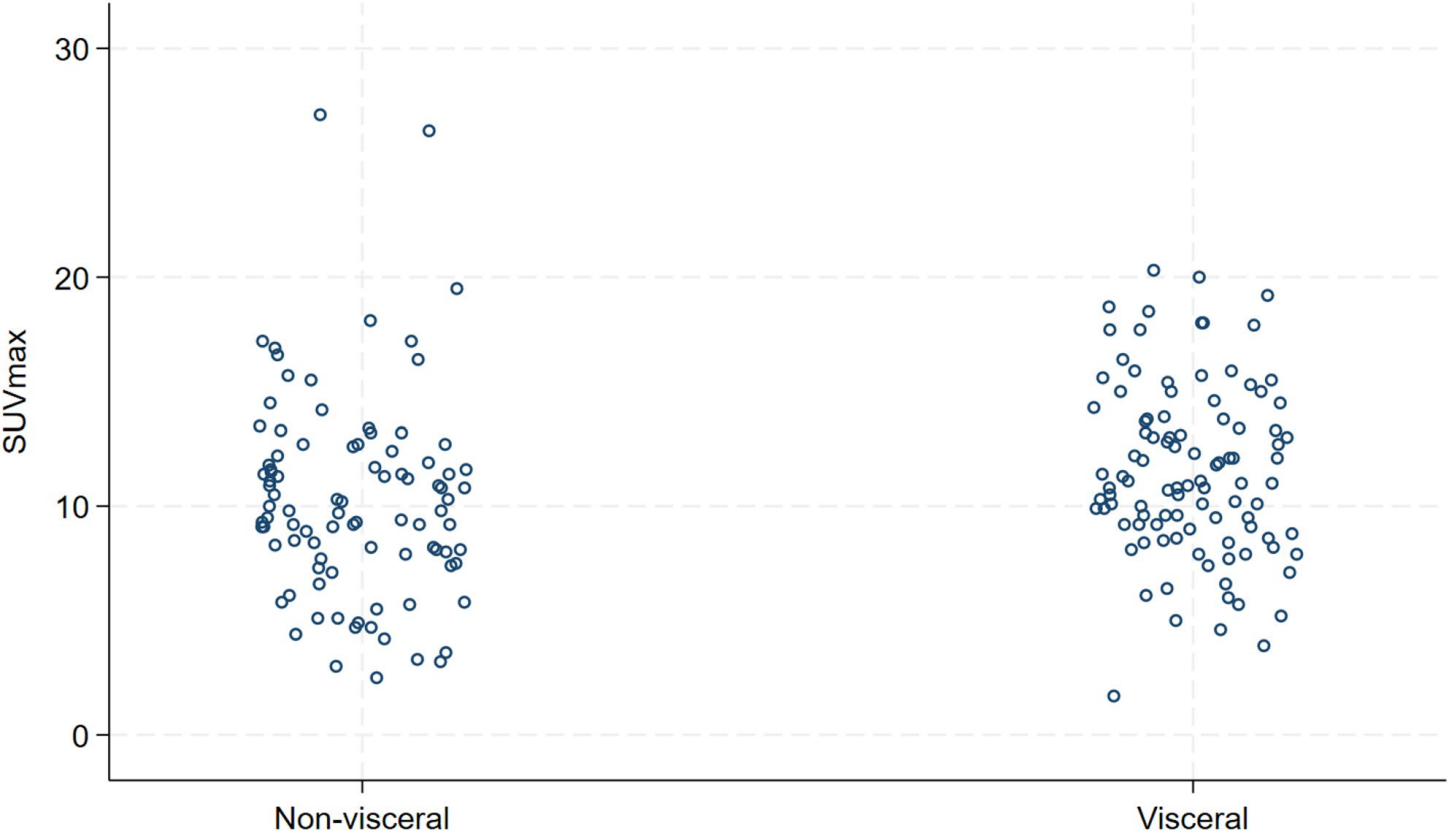
Distribution of baseline SUVmax by visceral involvement. Each dot represents an individual patient’s SUVmax value, stratified by the presence or absence of visceral metastases

A total of 194 patients were included in the analysis. The mean age was 58.9 years (range, 27.1–85.7). All patients were female, except for one male patient. De novo metastatic disease was diagnosed in 139 patients (71.7%). OMD, defined as ≤ 5 metastatic lesions, was present in 91 patients (46.9%). One hundred two patients (52.6%) were in ECOG PS 0, 85 in ECOG PS 1 (43.8%), and 7 in ECOG 2 (3.6%). Bone metastases were found at baseline in 112 patients (57.7%), 55 patients had liver metastases (28.4%), 52 patients had lung metastases (26.8%). There were two patients with brain metastases (1%), and both had SUVmax less than 9.6. Estrogen receptors (ER) were positive in 115 patients (59.3%), and negative in 79 patients (40.7%). All patients with ER-positive tumors received endocrine treatment as an addition to HP maintenance. The median follow-up from THP initiation was 38.1 months (IQR 22.8–57.9 months). The median time between FDG PET/CT and THP initiation was 0.9 months (IQR, 0.5 months − 1.6 months). Regarding histopathological subtype, 148 patients had no special type (NST, 76.3%), 8 patients lobular cancer (4.1%), 14 patients other specific subtype (7.2%), while for 24 patients (12.3%) the subtype was not described. A comparison between patients with high SUVmax (> 9.6) and low SUVmax (≤ 9.6) is presented in Table 1. The distribution of baseline SUVmax by disease extent (OMD vs. PMD) is shown in Fig. 1, whereas the distribution according to visceral involvement is presented in Fig. 2. The median follow-up was 37.5 months.

**Table 1:** Baseline characteristics of the study population and comparison between patients with low (≤ 9.6) and high (> 9.6) baseline SUVmax

Variable	Total (*n* = 194)	SUVmedian (range)	SUV ≤ 9.6 (*n* = 78)	SUV > 9.6 (*n* = 116)	*p*
Age, mean	58.9	10.8 (1.7–27.1)	58.5	58.9	0.945
ECOG 0	102 (52.6%)	10.8 (1.7–20.3)	41 (52.6%)	61 (52.6%)	0.230
ECOG 1	85 (43.8%)	10.8 (3.0-27.1)	32 (41.0%)	53 (45.7%)	
ECOG 2	7 (3.6%)	7.9 (3.3–13.3)	5 (6.4%)	2 (1.7%)	
De novo MBC	139 (71.7%)	11.0 (2.5–27.1)	53 (68.0%)	86 (74.1%)	0.417
OMD	91 (46.9%)	10.0 (3.2–26.4)	42 (53.9%)	49 (42.2%)	0.142
PMD	103 (53.1%)	11.0 (1.7–27.1)	36 (35.0%)	67 (65.0%)	
Bone metastases	112 (57.7%)	11.2 (2.5–27.1)	40 (51.3%)	72 (62.1%)	0.142
Liver metastases	55 (28.4%)	12.1 (5.7–20.3)	18 (23.1%)	37 (31.9%)	0.197
Lung metastases	52 (26.8%)	10.9 (1.7–18.7)	15 (19.2%)	37 (31.9%)	0.068
ER positive	115 (59.3%)	10.8 (1.7–26.4)	47 (60.3%)	68 (58.6%)	0.882

ECOG, Eastern Cooperative Oncology Group performance status; MBC, metastatic breast cancer; ER, estrogen receptor; OMD, oligometastatic disease; PMD, polymetastatic disease

### Progression-free survival

**Fig. 3:**
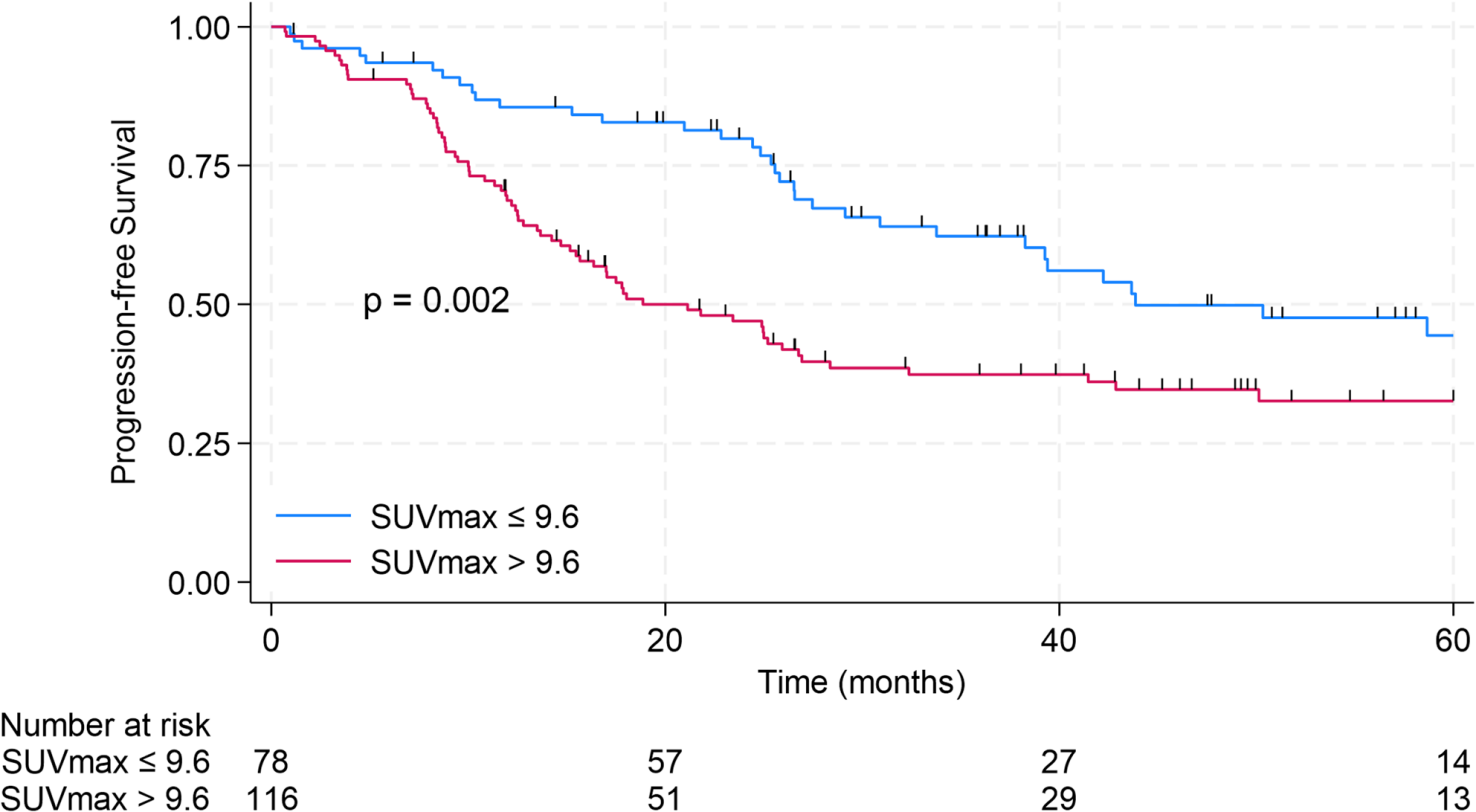
PFS stratified by baseline SUVmax (SUVlow vs. SUVhigh)

The median PFS for the entire cohort was 29.1 months, and 2-year PFS rate was 60.3% (95% CI 52.8–66.9%). Baseline SUVmax was significantly associated with PFS. When analyzed as a continuous variable, SUVmax was a prognostic factor for PFS (HR 1.06, 95% CI 1.01–1.11, *p* = 0.012). Each 1-unit increase in baseline SUVmax corresponded to a 6% higher risk of disease progression. The optimal SUVmax cutpoint for predicting PFS was 9.6 (sensitivity 0.8; specificity 0.53; area under the ROC curve 0.66). For further analysis, patients with SUVmax > 9.6 were classified as SUVhigh (*n* = 116), and those with SUVmax ≤ 9.6 as SUVlow (*n* = 78). The median PFS was 43.9 months in the SUVlow group versus 18.9 months in the SUVhigh group (*p* = 0.002). 2-year PFS was 79.8% (95% CI 68.8–87.3%) in SUVlow group and 47.0% (37.4–56.0%) in SUVhigh group (Fig. 3). In multivariate analysis, SUVmax, OMD, and ER positivity remained independent prognostic factors for PFS. Variables with *p* < 0.2 in univariate analysis are presented in Table 2. Age, liver metastases, and lung metastases were not significantly associated with PFS.

**Table 2:** Univariate and multivariate Cox proportional hazards regression analyses for PFS in the study cohort

Variable	Comparison	HR	[95% CI]	*p*
*Univariate analysis*				
ECOG	1 vs. 0	1.36	0.93–1.98	0.112
	2 vs. 0	0.88	0.21–3.62	0.860
ER	ER-positive vs. negative	0.55	0.38–0.80	**0.002**
OMD	OMD vs. PMD	0.59	0.41–0.87	**0.008**
SUVmax	> 9.6 vs. ≤ 9.6	1.89	1.27–2.81	**0.002**
*Multivariate analysis*				
ECOG	1 vs. 0	1.40	0.95–2.05	0.084
	2 vs. 0	0.71	0.17–2.96	0.643
ER	ER-positive vs. negative	0.47	0.32–0.70	**< 0.001**
OMD	OMD vs. PMD	0.61	0.41–0.89	**0.011**
SUVmax	> 9.6 vs. ≤ 9.6	1.98	1.32–2.98	**0.001**

HR, hazard ratio; ECOG, Eastern Cooperative Oncology Group performance status; ER, estrogen receptor; OMD, oligometastatic disease; PMD, polymetastatic disease. Statistically significant results are shown in bold

At the time of data cut-off, disease progression (PD) on THP treatment was documented in 102 patients. The most common sites of progression were the breast and/or regional lymph nodes (29/102), CNS (21/102), lungs (19/102), and liver (15/102).

### Overall survival

**Fig. 4:**
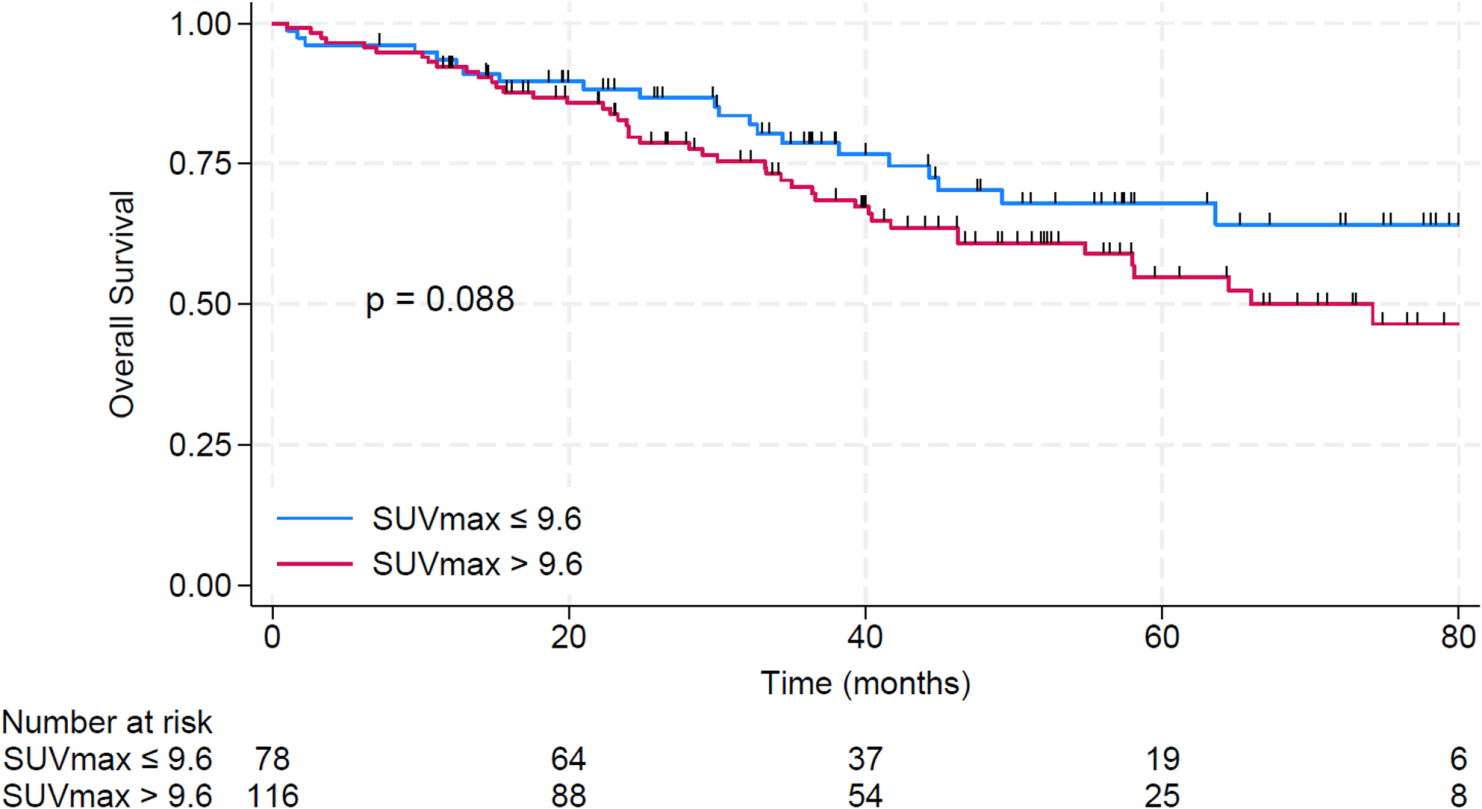
OS stratified by baseline SUVmax (SUVlow vs. SUVhigh)

The median OS for the entire cohort was 85.0 months. The 5-year OS rate was 60.1% (95% CI 51.1–67.8%). Baseline SUVmax, analyzed as a continuous variable, was not significantly associated with OS in univariate analysis. Median OS was not reached in the SUVlow group, compared with 74.2 months in the SUVhigh group (*p* = 0.088). The 5-year OS rate was 67.9% (95% CI 54.1–78.4%) in the SUVlow group versus 54.7% (95% CI 43.0–65.1%) in the SUVhigh group. (Fig. 4). In multivariate analysis, SUVmax (included as a dichotomous variable), OMD, and ER positivity remained independent prognostic factors for OS. Variables with *p* < 0.2 in univariate analysis are presented in Table 3.

**Table 3:** Univariate and multivariate Cox proportional hazards regression analyses for OS in the study cohort

Variable	Comparison	HR	[95% CI]	*p*
*Univariate*				
Age	Continuous	1.03	1.01–1.05	**0.008**
ECOG	1 vs. 0	1.79	1.09–2.92	**0.021**
	2 vs. 0	2.55	0.6–10.8	0.205
ER	ER-positive vs. negative	0.68	0.42–1.10	0.111
OMD	OMD vs. PMD	0.56	0.34–0.93	**0.024**
SUVmax	> 9.6 vs. ≤ 9.6	1.56	0.93–2.62	0.090
*Multivariate*				
Age	Continuous	1.02	0.999–1.05	0.052
ECOG	1 vs. 0	1.41	0.80–2.50	0.236
	2 vs. 0	1.69	0.38–7.50	0.489
ER	ER-positive vs. negative	0.57	0.34–0.95	**0.031**
OMD	OMD vs. PMD	0.53	0.31–0.88	**0.014**
SUVmax	> 9.6 vs. ≤ 9.6	1.46	0.87–2.46	0.152

HR, hazard ratio; ECOG, Eastern Cooperative Oncology Group performance status; ER, estrogen receptor; OMD, oligometastatic disease; PMD, polymetastatic disease. Statistically significant results are shown in bold

### Long-responders

**Fig. 5:**
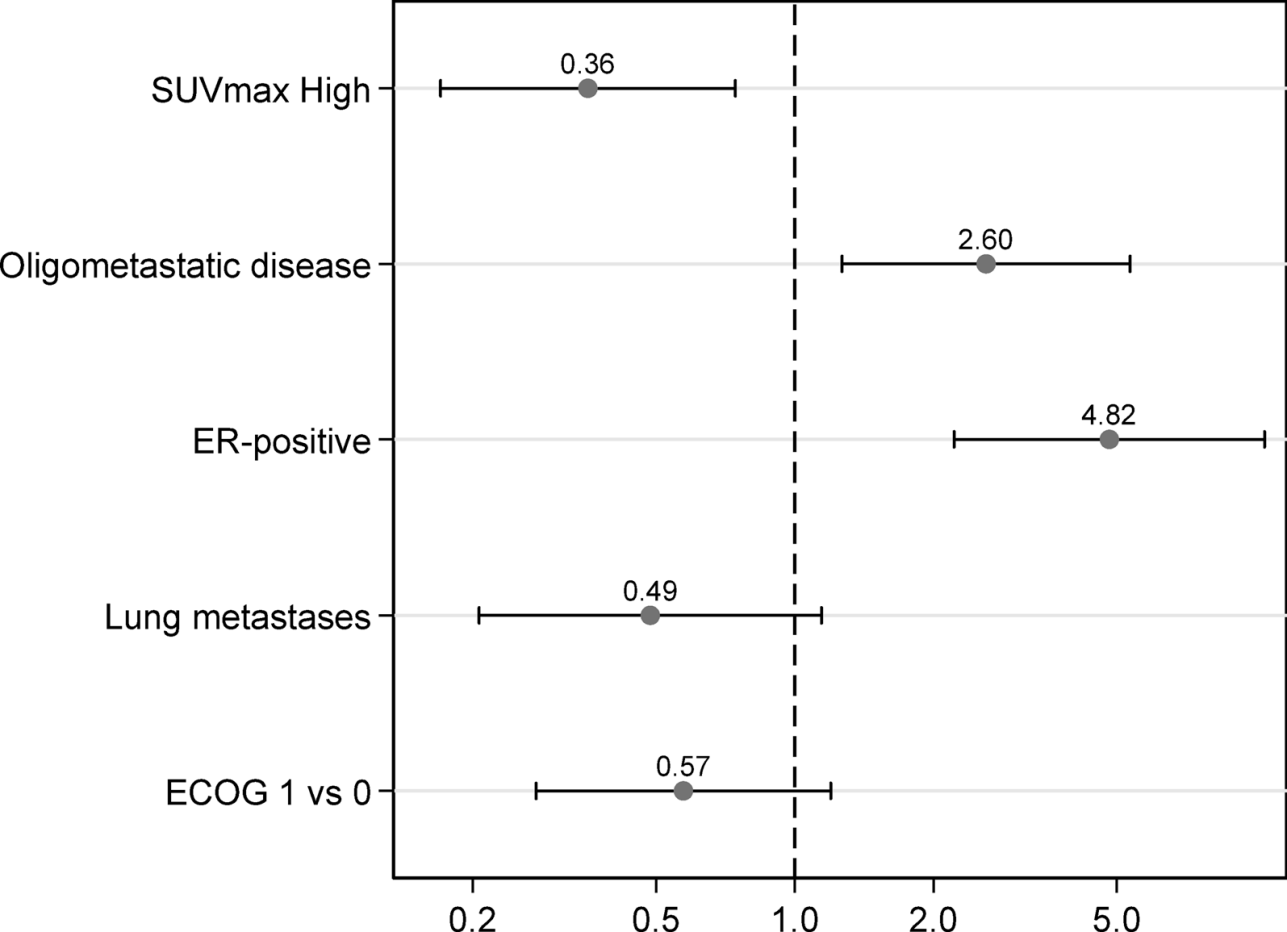
Multivariable logistic regression analysis of baseline predictors of long-term response (PFS ≥ 35 months) to first-line trastuzumab, pertuzumab, and docetaxel. The forest plot depicts odds ratios (ORs) for each variable included in the model

Among the 194 patients included in the overall cohort, 68 (35%) were classified as long responders, defined as PFS of 35 months or longer. A total of 94 patients (48%) had sufficient follow-up to be classified as non–long responders, while the remaining 32 patients (17%) were excluded from this subanalysis due to insufficient follow-up duration. Multivariable logistic regression analysis identified several baseline clinical variables associated with the likelihood of achieving long-term benefit from first-line trastuzumab, pertuzumab, and docetaxel therapy (Fig. 5). ER positivity was strongly associated with increased odds of long-term response (odds ratio [OR] 4.82; 95% confidence interval [CI] 2.22–10.46), as was the presence of OMD (OR 2.60; 95% CI 1.27–5.34). In contrast, a baseline SUVmax ≥ 9.6 was significantly associated with reduced odds of long-term benefit (OR 0.36; 95% CI 0.17–0.74). Although ECOG performance status 1 (vs. 0) and the presence of lung metastases were associated with numerically lower odds of durable response (OR 0.57; 95% CI 0.27–1.20 and OR 0.49; 95% CI 0.21–1.14, respectively), these associations did not reach statistical significance.

### Exploratory analysis: optimal SUVmax cut-off for overall survival

The optimal SUVmax cut-off for OS, calculated using ROC curve analysis and the Youden index, was 6.9 (sensitivity 0.93; specificity 0.25; AUC 0.59). For this exploratory analysis, patients with SUVmax > 6.9 were classified as SUVhigh (*n* = 166), and those with SUVmax ≤ 6.9 were classified as SUVlow (*n* = 28). Median OS was not reached in the SUVlow group, whereas it was 74.2 months in the SUVhigh group (*p* = 0.030). The 5-year OS rate was 82.1% (95% CI 59.0–92.9%) in the SUVlow group and 56.0% (95% CI 46.2–64.7%) in the SUVhigh group (Suppl Fig. 1). In the multivariable analysis, SUVmax (dichotomized by the 6.9 threshold), oligometastatic disease, and ER positivity remained independent prognostic factors for OS. Variables with *p* < 0.2 in univariate analysis are summarized in Suppl Table 1. To contextualize the differences between SUVmax thresholds identified for PFS and OS, among the 194 patients in the cohort, 28 (14.4%) had baseline SUVmax ≤ 6.9, and 116 (59.8%) had SUVmax > 9.6. The remaining 50 patients (25.8%) had SUVmax values between 6.9 and 9.6.

## Discussion

The PFS associated with THP treatment has improved over time, increasing from the median PFS of 18.5 months in the CLEOPATRA trial [4] to 26.9 months in the recently published interim analysis of the DESTINY-Breast09 study [7]. In our cohort, the median PFS was 29.0 months, consistent with these more recent outcomes. The 2-year PFS rate in our cohort was 60.3%, which exceeds the rate reported in CLEOPATRA (46%) and is slightly higher than in the THP arm of DESTINY-Breast09 [7]. This modest improvement may reflect differences in patient characteristics. In DESTINY-Breast09, 52% of patients had de novo metastatic disease and 54% had hormone receptor-positive (HR+) tumors [7]. In contrast, our cohort included a higher proportion of patients with de novo disease (71%) and a comparable proportion with HR+ status (59%).

However, the CLEOPATRA trial did not prospectively define or report a subgroup of patients with OMD, typically defined as the presence of up to five metastatic lesions [4]. Notably, the ESTRO–ASTRO consensus guidelines refrain from specifying a fixed numerical threshold, instead emphasizing that the number of lesions should be limited by the feasibility of delivering curative-intent metastasis-directed radiotherapy [28]. Consequently, no reliable data are available regarding the prevalence of OMD within the CLEOPATRA population. In contrast, a large real-world cohort from the National Cancer Database comprising 5376 women with de novo HER2-positive metastatic breast cancer reported that nearly half presented with a single metastatic site, and the overall metastatic burden was an independent predictor of survival [29]. However, the exact number of metastatic lesions was not detailed in that analysis. Importantly, a meta-analysis demonstrated that FDG PET/CT has significantly higher sensitivity for detecting distant metastases than conventional imaging in breast cancer patients [30]. In our cohort, all patients underwent baseline FDG PET/CT, which may explain the relatively high proportion (47%) classified as having oligometastatic disease (OMD), likely reflecting the superior sensitivity of PET/CT in detecting small or otherwise occult metastases that could have been missed by conventional imaging such as contrast-enhanced CT.

Multiple studies have demonstrated that patients with de novo OMD breast cancer experience longer OS. This observation is thought to reflect a combination of factors, including inherently slower tumor growth kinetics, limited metastatic potential, earlier detection leading to potential lead-time bias, and the frequent use of more aggressive and locally directed treatment strategies in this subgroup [31]. Patients with HER2-positive/HR-positive disease differ biologically from those with HER2-positive/HR-negative tumors, and some studies have reported an increased risk of early death in the HR-negative subgroup [32]. However, given the biological heterogeneity of HER2-positive breast cancer, intrinsic molecular subtypes provide more refined prognostic information than HR status alone [33]. Furthermore, the addition of CDK4/6 inhibitors to anti-HER2 therapy has been shown to further improve outcomes in selected patients [8, 34]. Consistent with these observations, in our study both OMD presentation and ER positivity remained independent prognostic factors associated with longer PFS.

Importantly, the distribution of baseline SUVmax values was similar between oligometastatic and polymetastatic disease (Fig. 1), indicating that the prognostic advantage of OMD cannot be explained by lower metabolic activity alone. Rather, SUVmax provides additional prognostic stratification within each disease extent category—identifying patients with more favorable outcomes among those with polymetastatic disease (lower SUVmax), as well as patients with poorer outcomes even within the oligometastatic subgroup (higher SUVmax). This supports the concept that baseline metabolic activity and disease extent capture complementary, non-overlapping aspects of tumor biology.

Guidelines strongly support the role of FDG PET/CT in baseline risk stratification, particularly in identifying patients with high disease burden who are unlikely to benefit from standard-duration first-line regimens [18]. In HER2-positive disease—which typically exhibits high FDG avidity—PET/CT improves the detection of distant and metastatic setting lesions and may inform early treatment intensification strategies. However, it is important to recognize that FDG uptake is significantly higher in invasive ductal carcinoma compared to invasive lobular carcinoma, which may limit sensitivity in certain histologic subtypes [35]. In our study, the majority of patients (76.3%) had invasive breast carcinoma of no special type (NST). Although the proportion of patients with invasive lobular carcinoma was small (4.1%), and other specific histologic subtypes comprised 7.2%, we chose not to exclude these patients. In our view, including these less common subtypes is important to enable future pooled analyses and multicenter studies focused on their imaging characteristics and treatment outcomes. For 24 patients (12.3%), the histologic subtype was not reported. However, we do not consider this a methodological shortcoming, as histological subtype classification relies on breast tumor biopsy. In cases where the biopsy was performed from a metastatic site, assessment of the primary tumor subtype may not have been feasible or reliable.

The end-of-study results of the CLEOPATRA trial showed that approximately 30% of patients in the pertuzumab group were classified as long-term responders (PFS ≥ 35 months) [36]. In our cohort, a comparable proportion (35%) met the criteria for long-term response (PFS ≥ 35 months), indicating that approximately one in three patients derived substantial and durable benefit from this treatment regimen. Moreover, after a median follow-up of 8 years, 16% of patients remained progression-free. These findings suggest that, in selected patients, maintenance therapy with trastuzumab and pertuzumab following initial THP induction may be sufficient to achieve sustained disease control, with approximately one in six patients maintaining long-term PFS [36]. In addition to the time-to-event Cox analysis, we performed a logistic regression using a clinically relevant binary endpoint—long-term responders were defined as patients still on PH maintenance treatment at 35 months, consistent with the long-term results from the CLEOPATRA trial [36]. This dual analytic strategy allowed us to examine not only which factors were associated with prolonged time to progression (hazard), but also to identify those independently associated with achieving durable benefit (odds). Notably, lower baseline SUVmax, presence of OMD, and luminal subtype emerged as independent predictors of long-term response. These factors were associated with more prolonged PFSand an increased chance of sustained benefit. This observation may have relevance for identifying patients more likely to benefit from standard first-line therapy and could support future work on biomarker-based treatment personalization in HER2-positive metastatic breast cancer.

In selecting first-line therapy for HER2-positive metastatic breast cancer, efficacy must be balanced with toxicity. The THP regimen (docetaxel, trastuzumab, pertuzumab) is primarily associated with hematologic toxicities, particularly neutropenia and febrile neutropenia (FN), which occurred in 13% of patients in the CLEOPATRA trial [4]. Notably, G-CSF prophylaxis was not mandated in the trial, likely contributing to FN rates. In current practice, however, G-CSF use has expanded, supported by biosimilars and updated ASCO, ESMO, and NCCN guidelines that recommend its use even in intermediate-risk settings. Real-world data on TCHP showed that primary G-CSF prophylaxis significantly reduced FN rates (2.4% vs. 18.5%) [37], suggesting that integrating G-CSF into THP regimens can similarly enhance safety. In contrast, T-DXd has a different toxicity profile. In DESTINY-Breast03, grade ≥ 3 adverse events occurred in 63.2% of patients, including ILD/pneumonitis in 16.7% [6]. While overall toxicity rates may appear comparable, THP-related toxicities are mainly hematologic, early, and self-limited, followed by a well-tolerated maintenance phase. T-DXd toxicities—particularly ILD—require ongoing surveillance, including serial chest CTs, as the drug is given until progression. Thus, THP with G-CSF support offers a more predictable and manageable toxicity profile, whereas T-DXd demands continuous risk mitigation throughout treatment.

Quantitative PET features—such as standardized uptake value (SUV), metabolic tumor volume (MTV), and total lesion glycolysis (TLG)—are valuable prognostic parameters and contribute to baseline staging [18]. These FDG PET/CT-derived metrics have demonstrated significant prognostic value in breast cancer, including metastatic disease [18]. A limitation of our study is that we assessed only SUVmax. Although widely used due to its simplicity and reproducibility, SUVmax reflects only the most metabolically active tumor area and fails to capture total tumor burden or intratumoral heterogeneity [38]. Whole-body TLG outperformed both SUVmax and receptor status as a predictor of OS in a cohort of 54 patients [38], while whole-body MTV was independently associated with shorter PFS in another study of 35 patients [39]. Moreover, nodal MTV, TLG, and SUVmax were significantly linked with recurrence risk in locally advanced breast cancer [40]. A meta-analysis of nine studies (975 patients) confirmed the prognostic significance of elevated MTV and TLG in the primary tumor [41]. Despite these data, their clinical use is limited by the need for post-processing software, standardized thresholds, and technical expertise that may not be universally accessible [42]. Thus, SUVmax remains the most practical and widely used PET parameter in both routine care and multicenter trials [43], with guidelines recommending its continued inclusion alongside volumetric metrics [18].

In our cohort, the median baseline SUVmax was 10.8, which is remarkably similar to values reported in the PHERGain study, where the median baseline SUVmax was 10.4 in a comparably sized population, supporting the representativeness of our dataset [44]. In PHERGain, patients achieving pCR had a lower baseline median SUVmax (8.8) compared with those without pCR (10.8). Importantly, PHERGain did not derive an optimal baseline SUVmax cut-off; instead, it used SUVmax—as in our study—rather than SULpeak, the parameter required by PERCIST criteria [45]. Consistent with our findings, another study also demonstrated higher baseline SUVmax among patients not achieving pCR compared with responders (11.2 vs. 7.9) [46]. Lower SUVmax thresholds (e.g., ≈ 3.0) have been reported in the early breast cancer (EBC) setting for predicting recurrence-free survival [47, 48], whereas other EBC studies proposed cut-offs around 4.9 for predicting residual axillary disease [49]. Collectively, these data highlight that SUVmax cut-offs are highly context-dependent and influenced by breast cancer subtype, disease stage (early vs. metastatic), clinical endpoints (pCR, RFS, PFS, OS), and treatment regimens. For these reasons, we emphasize that the cut-off of 9.6 identified in our study should be interpreted within the specific context of HER2-positive metastatic breast cancer receiving first-line THP. While dichotomization facilitates Kaplan–Meier visualization and clinical interpretability, it inevitably simplifies a continuous biological measurement. Indeed, in our analysis, modeling SUVmax as a continuous variable provided additional insight: each 1-unit increase in baseline SUVmax was associated with a 6% higher risk of disease progression. This underscores that the prognostic effect of tumor metabolism is not a binary phenomenon, but rather a gradient reflecting underlying tumor biology.

We acknowledge several limitations of our study. First, its retrospective design inherently introduces potential biases and limits causal inference. Additionally, the majority of our cohort comprised patients with de novo metastatic disease, which likely reflects the increased use of FDG PET/CT as a staging tool that facilitates early detection of metastatic lesions, thereby reclassifying some patients as de novo MBC. PIK3CA mutation status was not assessed in our cohort, as it is currently under investigation in prospective clinical trials and is not yet incorporated into routine clinical decision-making for HER2-positive MBC. Some heterogeneity in the baseline population may also be considered a limitation. We chose not to exclude patients with brain metastases; however, only two such cases were present, and both were in the low SUVmax group, thus not contributing to the poorer outcomes observed in the high SUVmax group. While the majority of patients had tumors of no special type (NST), the inclusion of cases with invasive lobular carcinoma and other specific histological subtypes reflects real-world clinical practice.

We recognize that imaging biomarkers alone may not capture the full biological complexity of HER2-positive MBC. Molecular assays appear more promising in this regard. In particular, tests such as the HER2DX assay, which includes the ERBB2 score, have demonstrated prognostic utility—showing significant differences in PFS based on ERBB2 expression levels, both in the CLEOPATRA trial and in real-world evidence datasets [50]. Future studies should aim to refine and integrate molecular and imaging biomarkers to enable more precise treatment stratification in HER2-positive MBC. The combination of advanced molecular profiling with functional imaging may be especially valuable in this context.

Our findings demonstrate that baseline metabolic activity on FDG PET/CT, particularly SUVmax, has independent prognostic value in HER2-positive metastatic breast cancer treated with first-line trastuzumab, pertuzumab, and docetaxel. High SUVmax was associated with shorter PFS and OS, while low SUVmax, ER positivity, and OMD predicted long-term benefit. These results support the role of SUVmax as a noninvasive, accessible biomarker to inform risk-adapted treatment strategies. As first-line options expand to include antibody–drug conjugates and biomarker-driven approaches, functional imaging may complement molecular profiling to improve patient stratification. Further prospective research is warranted to explore progression patterns in patients with high SUVmax and to evaluate how tools like HER2DX can be integrated into predictive models for individualized treatment planning.

## Electronic Supplementary Material

Below is the link to the electronic supplementary material.

Supplementary Material 1.

## Data Availability

The datasets generated and/or analyzed during the current study are not publicly available due to confidentiality reasons but are available from the corresponding author on reasonable request.

## Abbreviations

ACR: American College of Radiology
CDK4/6: Cyclin–dependent kinases 4 and 6
CE: CT–Contrast–enhanced computed tomography
CNS: Central nervous system
EANM: SNMMI–European Association of Nuclear Medicine–Society of Nuclear Medicine and Molecular Imaging
EBC: Early breast cancer
ECOG: Eastern Cooperative Oncology Group
ER: Estrogen receptor
ESSO: European Society of Surgical Oncology
ESTRO: European Society for Radiotherapy and Oncology
EUSOBI/ESR: European Society of Breast Imaging/European Society of Radiology
EUSOMA: European Society of Breast Cancer Specialists
FDG PET/CT: 2–[¹⁸F]fluoro–2–deoxy–D–glucose positron emission tomography/computed tomography
HER2: Human epidermal growth factor receptor 2
HR: Hormone receptor
IQR: Interquartile range
MBC: Metastatic breast cancer
OMD: Oligometastatic disease
OR: Odds ratio
OS: Overall survival
PD: Progressive disease (disease progression)
PFS: Progression–free survival
PS: Performance status
ROC: Receiver operating characteristic
SUVmax: Maximum standardized uptake value
T-Dxd: Trastuzumab deruxtecan
THP: Docetaxel, trastuzumab, and pertuzumab regimen
