# Supplementary material for: Prognostic value of baseline FDG PET/CT in HER2-positive metastatic breast cancer treated with first-line trastuzumab, pertuzumab, and docetaxel
Source: Breast Cancer Res. 2025 Dec 10;28:32. doi: 10.1186/s13058-025-02191-7 (PMC12866219; doi:10.1186/s13058-025-02191-7)
Supplement: Supplementary file 1 — Supplementary Material 1. [file 13058_2025_2191_MOESM1_ESM.docx]

**Figure 1** (Supplementary). Kaplan–Meier overall survival curves stratified by the exploratory SUVmax cut-off of 6.9.

| Univariate | | | | |
| --- | --- | --- | --- | --- |
| Variable | **Comparison** | **HR** | **[95% CI]** | **p** |
| Age | continuous | 1.03 | 1.01-1.05 | **0.008** |
| ECOG | 1 vs 0 | 1.79 | 0.92-1.94 | **0.021** |
|  | 2 vs 0 | 2.55 | 0.6-10.8 | 0.205 |
| ER | ER-positive vs negative | 0.68 | 0.42-1.10 | 0.111 |
| OMD | OMD vs PMD | 0.56 | 0.34-0.93 | **0.024** |
| SUVmax | >6.9 vs ≤6.9 | 2.64 | 1.06-6.57 | **0.037** |
| Multivariate | | | | |
| Variable | **Comparison** | **HR** | **[95% CI]** | **p** |
| Age | continuous | 1.02 | 0.999-1.05 | 0.056 |
| ECOG | 1 vs 0 | 1.44 | 0.81-2.55 | 0.210 |
|  | 2 vs 0 | 1.99 | 0.44-9.05 | 0.373 |
| ER | ER-positive vs negative | 0.59 | 0.36-0.98 | **0.043** |
| OMD | OMD vs PMD | 0.54 | 0.33-0.90 | **0.019** |
| SUVmax | >6.9 vs ≤6.9 | 2.53 | 1.01-6.34 | **0.047** |

Abbreviations: HR – hazard ratio; ECOG – Eastern Cooperative Oncology Group performance status; ER – estrogen receptor; OMD – oligometastatic disease; PMD – polymetastatic disease. Statistically significant results are shown in bold.

**Table 1** (Supplementary). Univariate and multivariable Cox proportional hazards analyses for overall survival using the exploratory SUVmax cut-off of 6.9.
